# Contained Power Morcellation in Laparoscopic Uterine Myoma Surgeries: A Brief Review

**DOI:** 10.3390/healthcare11182481

**Published:** 2023-09-07

**Authors:** Bogdan Obrzut, Marta Kijowska, Marzanna Obrzut, Adam Mrozek, Dorota Darmochwał-Kolarz

**Affiliations:** 1 Department of Obstetrics and Gynecology, Institute of Medical Sciences, Medical College, University of Rzeszów, Rejtana 16 C, 35-959 Rzeszow, Poland; 2 Department of Obstetrics and Gynecology, Provincial Clinical Hospital No. 2 Rzeszow, Lwowska 60, 35-301 Rzeszow, Poland; 3 Institute of Health Sciences, Medical College, University of Rzeszow, Warzywna 1a, 35-310 Rzeszow, Poland

**Keywords:** contained morcellation, retrieval bag, tissue dissemination, myoma, laparoscopy

## Abstract

Uterine fibromas are the most common benign uterine tumors. Although the majority of leiomyomas remain asymptomatic, they can cause serious clinical problems, including abnormal uterine bleeding, pelvic pain, and infertility, which require effective gynecological intervention. Depending on the symptoms as well as patients’ preferences, various treatment options are available, such as medical therapy, non-invasive procedures, and surgical methods. Regardless of the extent of the surgery, the preferred option is the laparoscopic approach. To reduce the risk of spreading occult malignancy and myometrial cells associated with fragmentation of the specimen before its removal from the peritoneal cavity, special systems for laparoscopic contained morcellation have been developed. The aim of this review is to present the state-of-the-art contained morcellation. Different types of available retrieval bags are demonstrated. The advantages and difficulties associated with contained morcellation are described. The impact of retrieval bag usage on the course of surgery, as well as the effects of the learning curve, are discussed. The role of contained morcellation in the overall strategy to optimize patient safety is highlighted.

## 1. Introduction

Uterine leiomyomas [fibroids] are the most commonly diagnosed form of benign gynecological tumors, consisting of smooth muscle and abnormal dysfunctional extracellular matrix. Although uterine myomas occur in up to 80% of women of reproductive age [1,2,3,4], the majority of them remain asymptomatic. However, 20–40% of patients with fibroids demonstrate severe clinical problems (e.g., abnormal menstrual bleeding and bleeding between periods, pelvic pain, dysmenorrhea, infertility, recurrent pregnancy loss, or other complications during gestation) [2,5,6,7,8]. The consequences of such problems include a significant deterioration of psychological well-being, reduced physical activity, limited work productivity, as well as impairment of both intimate and social relationships [4]. Hence, symptomatic uterine leiomyomas require effective gynecological treatment.

For women who wish to avoid surgery, contemporary gynecology offers numerous treatment options, including hormonal therapy and antifibrinolytic or non-steroidal anti-inflammatory drugs [8,9,10]. For symptomatic women, following the failure of medical treatment, non-invasive procedures are proposed [8]. Uterine fibroid embolization is a non-surgical treatment option where the blood supply of a myoma is blocked through an embolizing agent applied intravascularly by an interventional radiologist [11,12]. Another non-invasive therapy is magnetic-resonance-guided focused ultrasound surgery/ablation. The ultrasound transducer delivers focused sound waves, which heat and destroy fibroid tissue leading to necrosis [13,14,15]. The next non-invasive treatment is transcervical, intrauterine sonography-guided radiofrequency ablation. Thermal energy is applied through a needle electrode placed in the myoma to induce coagulative myolysis [8].

For symptomatic women, after ineffective medical and/or non-invasive treatment, surgical procedures are offered [16]. Surgical treatment options depend on the patient and fibroid characteristics and include myomectomy (in patients who want to preserve their fertility) or hysterectomy (in patients who are beyond childbearing) [16]. To remove the fibroids, different approaches can be taken: hysteroscopic, laparoscopic (including robotically assisted), or traditional laparotomy. For hysterectomy, a vaginal, laparoscopic (including robotic), or open approach can be chosen.

Keeping in mind the prevalent symptoms and the preferences of the patient, the final decision on the selection of treatment modality is guided by imaging techniques, mainly ultrasound examination and magnetic resonance imaging [4]. Ultrasonography is usually sufficient for confirmation of the initial diagnosis but is insufficient in the assessment of the leiomyoma’s viability and blood supply, parameters that are crucial for the prediction of the possible effectiveness of uterine artery embolization (UAE) and/or magnetic-resonance-guided focused ultrasound ablation (MRgFUS/HIFU) [16]. Magnetic resonance imaging (MRI) is significantly more accurate for leiomyoma mapping and leiomyoma viability assessment, as well as the evaluation and differentiation of coexisting uterine pathology.

Despite the relatively wide availability of the numerous, above-listed conservative management options, surgical procedures still remain the basic treatment form for uterine fibroids. The spectacular development of laparoscopic technology has led to the vast majority of leiomyoma open operations being carried out via laparoscopy. This change in surgical approach is supported by the well-known advantages of laparoscopy, such as minimal traumatizing of the abdominal wall, excellent cosmetic effect, and a quick return to health and full physical activity. Except for cases of total laparoscopic hysterectomy followed by transvaginal removal of the uterus, all the remaining laparoscopic operations related to leiomyomas include sharp fragmentation of the specimen. This technique, called power morcellation, was introduced by Steiner and first described in 1993 [17]. Since then, many types of morcellators have been developed and have become an integral element of the laparoscopic instrumentarium. Recently, mechanical intraperitoneal morcellation has become the subject of heated discussion because of the risk of intraabdominal dissemination of preoperatively undiagnosed malignant tumors [18,19]. The true incidence of uterine sarcoma developing from fibroma remains unknown. In myomectomy or hysterectomy specimens, sarcomas were detected in 0.02–0.8% of women [20,21,22], while within morcellated specimens, they were detected in 0.02% of cases [23]. The probability of morcellation of occult neoplastic tissue during laparoscopic myomectomy or hysterectomy varies from 0.28 to 0.52% [24,25,26]. However, the accurate evaluation of the risk of an accidental morcellation of the leiomyosarcoma is challenging because of the paucity of research [27,28,29,30,31]. It is equally difficult to assess the real impact of morcellation on a patient’s prognosis. The prospective randomized trials necessary for this purpose are impossible to conduct for bioethical reasons. Nonetheless, the scientific data from previously published retrospective cohort studies unequivocally show that morcellation of stage I occult sarcoma involves a very high risk of its intraabdominal dissemination [23,27,28]. Furthermore, it relates to a higher recurrence rate and worsening of the patient’s prognosis [32,33,34]. Unfortunately, none of the currently available diagnostic tools are able to reliably detect an occult neoplastic tissue within a uterine fibroma. In 2014, Nagai et al. developed the PRESS algorithm (PREoperative Sarcoma Score) [35], which was revised in 2015 as rPRESS [36]. It aimed to increase the percentage of preoperative diagnoses of malignancies within the uterus and take appropriate therapeutic actions. It was based on the detection and evaluation of risk factors for uterine malignancy: patient’s age, LDH level in plasma, MRI result, and endometrial biopsy results. The diagnostic value of this algorithm is 84.1%, and its sensitivity and specificity are 80.0% and 85.4%, respectively. The European Society of Gynaecological Oncology identified additional risk factors, such as ethnicity (Afro-American ethnicity is an aggravating circumstance), status post pelvic irradiation (radiotherapy), tamoxifen use, family history of cancer (retinoblastoma), and a rapid growth of the mass, especially in postmenopausal patients [37]. Unfortunately, neither the above-cited studies, nor the ensuing research, have made a substantive breakthrough, and pretreatment differential diagnosis between common fibroma and uterine sarcoma remains very challenging [38,39,40,41,42,43,44]. Consequently, in 2014, the US FDA discouraged the routine use of laparoscopic power morcellators for removal of uterus or uterine leiomyomas [45]. Apart from malignant tumor dissemination, parasitic leiomyomatosis and iatrogenic peritoneal adenomyosis (smooth muscle hyperplasia with endometrial glands) resulting from tissue spillage have also been reported [46,47,48,49,50,51,52,53]. According to the review by Van der Meulen, the median time from the operation to recognition of parasitic leiomyomatosis was 48 moths [54]. In contrast, symptomatic peritoneal adenomyosis can be diagnosed as early as 6 months after laparoscopic supracervical hysterectomy with uncontained morcellation [55,56,57]. The overall risk of the development of iatrogenic peritoneal endometriosis after LASH with uncontained morcellation is assessed to be between 0.57 and 1.4% [48,58]. However, some experts say that the true incidence of this iatrogenic complication could be higher [59].

Considering these facts, and the FDA’s warning, operating gynecologists faced a real decisional dilemma because the undeniable advantages of minimal-access surgery could not be overlooked [60]. Additionally, converting laparoscopic cases to laparotomy correlates with an increased risk of morbidity and mortality [61]. A decision analysis showed that with open hysterectomy, there would be more overall deaths than with laparoscopic hysterectomy (103 vs. 98 per 100,000). This statistic remains stable even when including deaths from leiomyosarcoma in the laparoscopic patients [25]. In response to the FDA’s statements, to ensure patient safety and simultaneously maintain the acknowledged advantages of laparoscopic approach, a new strategy to remove the surgical specimen from the abdominal cavity was proposed. It resulted in the development and implementation of various systems for tissue extraction and the replacement of the traditional technique with so-called contained or in-bag morcellation [53,62,63,64,65,66].

The aim of this brief review is to offer a comprehensive update on contained laparo-scopic morcellation and to indicate future challenges. We focus on practical aspects of bag use and its impact on the course of the operation.

A comprehensive search was conducted on PubMed (2014–2023) and Scopus (2014–2023). Several keywords and their combinations were used to retrieve articles from these databases, including contained morcellation, in-bag morcellation, power morcellation, endobag, myomectomy, hysterectomy, leiomyosarcoma, and malignancy dissemination. Articles were included if they were cohort, clinical, case–control studies, or case series. Case reports and studies involving animals were excluded.

## 2. State of the Art

Laparoscopic morcellation with the use of a retrieval bag was first reported by urologists in 1993 [67]. Subsequent years brought a further spread of this technique in the field of urology, which was used for nephrectomy for early-stage, low-grade clear cell cancer. From a technical perspective, bags were introduced into the operating field through a port side and then, after putting the specimen into it, pulled out. The morcellation (mechanical or electrical) was conducted under direct visual control or with the help of a telescope, which was introduced into the bag via its exteriorized mouth beneath the morcellator [68,69,70,71]. The primary goal of the application of contained morcellation in such cases was the prevention of the iatrogenic spillage of neoplastic cells, and especially avoiding port-site metastases. However, the oncologic results of the described technique were controversial [68,72,73]. A similar approach was also described by general surgeons. The first indication was splenectomy with subsequent manual morcellation of the specimen in an exteriorized retrieval bag [74].

The history of in-bag morcellation in gynecological surgery is also quite rich. To date, various techniques have been reported, which differ from each other in terms of indication, installation of the bag, type of morcellation, and ways of removing tissue. As far as approach is concerned, bags were introduced through the abdominal port site, transvaginally, or via laparotomic incision [75,76,77,78]. Vaginal in-bag morcellation became relatively popular [79,80,81]. After the US FDA statement, numerous systems for laparoscopic extraction of the specimen from the peritoneal cavity have been designed and implemented (e.g., EndoCatch bag, Covidien; Anchor TRS-200, Anchor Surgical; LapSac Surgical Tissue Pouch, Cook Medical; and Steri-Drape Isolation Bag, 3M). The above-listed containers were made of different materials and were of various sizes; however, they were of a comparable shape, consisting of a sac with one large opening. They allowed mechanical fragmentation of the specimen using a morcellator inserted into the bag through its exteriorized mouth and removal of tissue fragments without contact with the peritoneal cavity. If direct visual control was impossible or insufficient, laparoscopic guidance was necessary. Cohen [75] described a single-incision umbilical access for both the morcellator and the telescope which in fact was analog to the established vaginal approach. In the case of multiport laparoscopy, at least one intraperitoneal puncture of the insufflated bag containing a specimen was required to introduce the optic. This was a crucial weak point of all these first-generation systems, due to the potential risk of intraabdominal tissue dissemination.

Currently, the most frequently used are the second-generation containers, called two-port or dual opening bags. They are usually made of polyurethane or polyurethane-coated nylon fabric [62,64,65]. This material is inflatable, impermeable for cells and water, not inflammable, and resistant to thermal effects caused by cold light exposure. The majority of the bags are transparent, which increases the safety of the procedure. Transparency allows simultaneous visual control of the bag content and the surrounding abdominal structures. Thanks to this bag wall property, the operating surgeon can continue the morcellation while being perfectly aware of the neighboring tissues and maintaining the appropriate protective distance from them [62]. The contemporary bags are rectangular or stomach shaped. Their dimensions are defined by the anatomy of the human abdomen. They are offered in different volumes (mainly between 1.6 and 4 L) and should be chosen according to the size of the patient and the surgical specimen.

**Figure 1:**
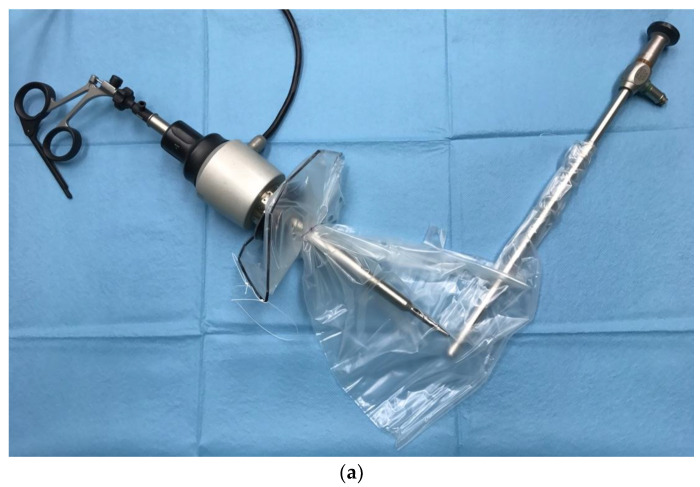

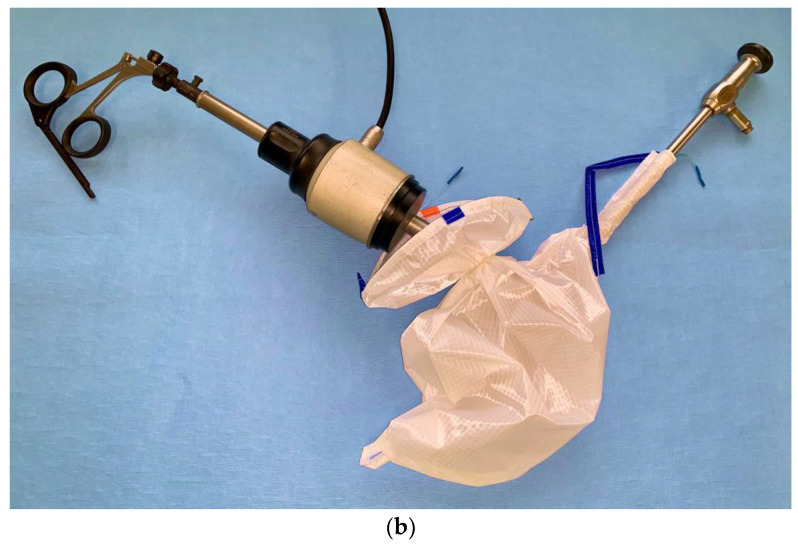
Exemplary bags for contained morcellation: (**a**) More-Cell-Safe A.M.I. GmbH, Feldkirch, Austria; (**b**) EMP200ECO-TMF-6, Fannin Ltd., Somerset, UK.

The typical dual opening bag has two separate ports (Figure 1).

**Figure 2:**
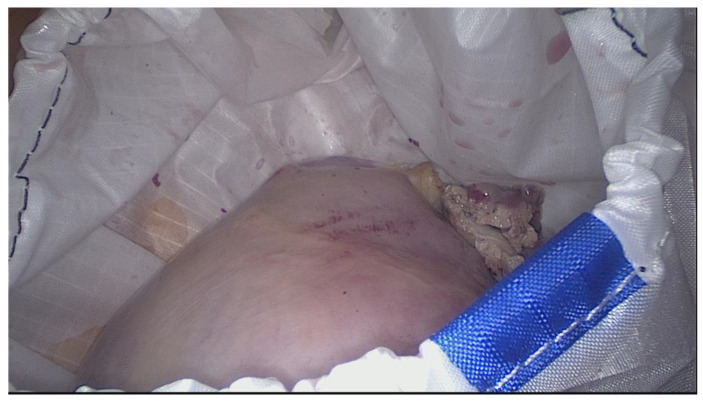
Uterine specimen in the bag inside the peritoneal cavity.

The diameter of the main opening varies between 13 and 17 cm, depending on the size of the bag and the manufacturer. This opening is used to put a specimen into the bag, which might be myoma/myomas, corpus uteri, the entire uterus, etc., according to conducted procedure. The large opening also serves as morcellator access. The second port (opening) is designed for the insertion of the optic trocar at the umbilical site. It is sleeve-shaped, narrow (about 15–17 mm), and long (10–19 cm). In some of the systems, the second opening (second sleeve) is double-layered [62,63]. After the morcellation is completed and the telescope removed, the sleeve is turned inside out and closed by tying a double knot. This means that the potentially contaminated part of the sleeve that comes into contact with the abdominal organs during removal of the bag remains oncologically “clean”. After the surgical phase of the procedure is completed, the bag is inserted into the abdominal cavity through a left lateral or suprapubic trocar with a 12–15 mm diameter. Once inside the peritoneal cavity, the bag is unrolled and opened by blunt graspers to prevent any damage to its integrity. In some systems, the bag is rolled up beforehand and placed in a specially designed cover, which can ease and shorten this phase of the procedure. Other manufacturers offer folded bags which allow for self-opening. After opening the large mouth, the specimen is placed into the bag (Figure 2) with the help of grasping forceps using the laterals or alternatively suprapubic and right trocars.

**Figure 3:**
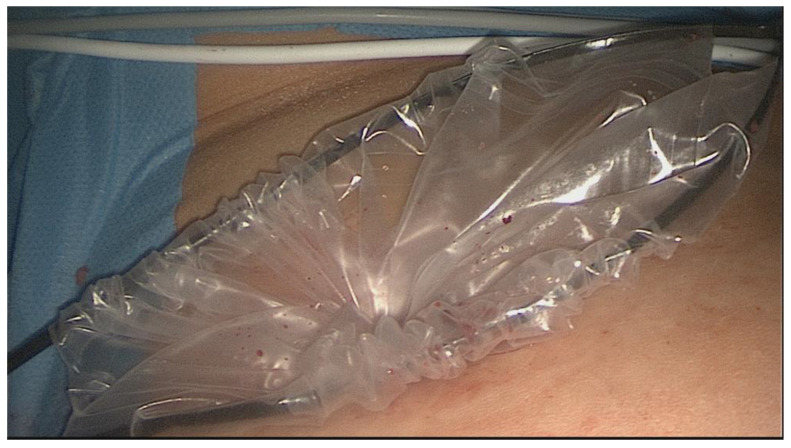
The large opening of the retrieval bag exteriorized for morcellator access.

Once the specimen is positioned, the large opening is closed by pulling the drawstring and extracted outside the abdominal wall (Figure 3).

The second opening is pulled out through the umbilical access while the previously inserted optic trocar is removed. At this point, the recently removed optic trocar is reinserted into the bag via the small tubular opening and so-called pseudoperitoneum is established by inflating with CO_2_. Next, the morcellator is entered bluntly into the bag through its large opening. The power morcellation proceeds in a contained space, but its technique remains unchanged. Once the morcellation is completed, the electro-morcellator, as well as the optic, are removed from the bag. Small pieces of specimen and a certain volume of blood remain in the bag. Depending on the study, the median weight of the bag content varies from 12 to 29 g [82,83]. This amount of residual fluid with tissue fragments does not hinder the correct bag removal. After securing the small opening, the bag is slowly extracted through the left lateral or suprapubic access. A very important step is checking the bag walls in terms of their tightness by thorough visual control and fluid filling. For final inspection of the operating field in terms of bleeding or potential organ complications, the pneumoperitoneum should be reestablished, and the optic trocar reinserted. It is crucial to know that the telescope, previously used inside the bag and the optic trocar, can be contaminated by fluid and tissue fragments originating from specimen morcellation. Thus, a new optic as well as new optic trocar should be utilized to perform the final inspection of the operating field. Another solution is to use a special optic cover during the in-bag morcellation, offered by some manufacturers, protecting the telescope from contamination. This accessory element of the instrumentarium is discarded after completion of morcellation [62,63].

Recent years have brought a new solution in the field of closed morcellation, the so-called third-generation bag, which is a multiport retrieval container. Apart from a large opening, it is equipped with three working ports, through which instruments of a diameter up to 15 mm can be inserted, for example graspers or irrigator. The large mouth is self-opening. Sealable access points allow safe retrieval of the bag. Multiport bags are not yet widely spread, and publications relating to their use are lacking.

## 3. Impact of the Bag Use on the Course of the Operation

Introducing the new bag systems to the routine surgical practice was preceded by thorough preclinical evaluation of its ability to diminish the risk of fluid and cell dissemination during the surgical procedures. The properties of the bag material were first tested in vitro, where the morcellation of animal tissue in a laparoscopy training set was performed with the usage of typical laparoscopic instrumentarium [62]. As a next step in preclinical testing, in vivo experimental evaluation was conducted in an animal model on farmhouse pigs [62].

To objectively assess the practical utility of a retrieval bag and the impact of contained morcellation on the entire course of the operation, numerous aspects of the procedure and its specific phases must be taken into consideration. They involve in particular:

- Insertion of the retrieval bag into the peritoneal cavity;
- Deployment of the bag;
- Positioning of the bag inside the abdominal cavity;
- Positioning the specimen into the bag;
- Large-opening exterioration;
- Exterioration of the optic sleeve;
- Insertion of the umbilical trocar;
- Establishing the pseudoperitoneum;
- Introduction of the telescope into the inflated bag;
- Initial and continuous in-bag visualization;
- Insertion of the morcellation device;
- Manipulation of the specimen within the bag to find the optimal position for morcellation;
- Course of the in-bag morcellation;
- Morcellation-associated complications;
- Uncontaminated closing of the optic sleeve;
- Removal of the bag;
- Damage of the containment bag;
- Occurrence of any organ damage related to bag use;
- Other intraoperative complications;
- Time necessary to conduct particular steps of the procedure;
- Overall satisfaction of the operating doctor.

Generally, numerous publications have confirmed in-bag morcellation to be feasible and satisfactory [64,65,66,82,84]. A pilot study conducted by Rimbach et al. revealed that from the technical point of view, the use of the dual-opening contained bag was successful in the vast majority of procedures. Only in one case did the intended use of the bag fail, because the size of the specimen turned out to surpass the diameter of the bag mouth [63]. An additional total time related to bag use ranged from 8.5 to 26.5 min (median 14 min). Introduction of the bag and its deployment lasted 1.5 ± 1 min, placement of the specimen 4.7 ± 5.7 min, extraction of the bag’s large opening 2.7 ± 1 min, entering the optic trocar while establishing the pseudoperitoneum 5.6 ± 4.3 min, inserting the morcellation device 1.3 ± 0,7 min, and closing and removing the containment bag 1.9 ± 0.7 min [63]. Subsequent studies on contained morcellation with the application of dual-opening systems reported the time related to bag use as ranging from 7 to 22 min [84,85,86,87]. Other reports revealed that contained morcellation prolongs the total time of the operation by about 20–30 min compared to the retrospective control group [76,78,88]. However, the specimen diameter in the contained morcellation cohort was greater than in the controls, which itself could make the certain steps of the procedure more challenging and thus longer lasting. Apart from the specimen size, the time related to bag use is influenced by many other factors such as experience and individual skills of the surgeon, positioning of the patient, and their weight [59].

In-bag morcellation, as any other new operating technique, has its own learning curve. Observations demonstrate that systematic training allows for a reduction in the median total time related to bag use from 14 to 10 min. Another positive aspect of the learning curve is the growth in the technical success rate, which is reported to increase from 85.7% to 93.9% [63,82]. While the technical success is usually defined as realization of the plan and achieving the aim of the operation, in the case of contained morcellation, the objective criterium of a successful procedure is the absence of smooth muscle cells in the perineal washing after completing the procedure and bag removal. Only this criteria confirms the efficient prevention of dissemination of cells from the extirpated and morcellated specimen. In the above-cited studies, the cytological examination of the peritoneal fluid did not reveal smooth muscle cells in any of the patients [63,82].

As shown above, the laparoscopic contained morcellation technique requires additional time for bag use. However, this approach also saves time, because the meticulous collection of tissue remnants and extensive washing of the abdominal cavity, typical for uncontained morcellation, is not necessary. The study of Krentel et al. demonstrated that this effect can be spectacular. According to these authors, after one year of use of the retrieval bag, the duration of the entire procedure with in-bag morcellation, from first skin incision to the last suture, does not differ from its counterpart with uncontained morcellation. What is more, after 2 years of regular practice, the total surgical time with use of the bag is significantly shorter than in a control group [59].

Another advantage of using the retrieval bag is the walls of the inflated bag pushing up intestinal slings. This makes the operating space more comfortable for the surgeon and simultaneously safer for the patient.

Despite the relatively high success rate, difficulties with handling of the bag happen in about 16% of cases. A too small umbilical fascia incision may cause problems with optic trocar placement into the optic sleeve, which leads to repeated attempts of insertion or additional enlarging of the fascia opening. Sometimes the bag remains twisted after insertion and requires removal with subsequent replacement [82].

According to published studies, the weight of the surgical specimen subjected to contained morcellation varied from 18 to 2805 g [63,64,65,82,87]. The size of the specimen can be a real limitation of the use of the retrieval bag. This may hinder the specimen placement and its positioning in the bag as well as the handling of the morcellator and/or the telescope. It is obvious that large specimens require bags of greater volume. However, there is always a question of finding a compromise between obtaining more space for in-bag morcellation and making the insertion and preparation of the bag within the peritoneal cavity more challenging.

The risk of bag damage with subsequent tissue dissemination depends on the type of tissue extraction system, applied surgical technique, experience of the operating team, and the size of the specimen. Early studies reported damage to the bag in about 6% of cases [82]. This happened mainly during optic trocar insertion, through handling of morcellator forceps, or during forced extraction of the bag containing bigger tissue remnants [82]. Gaining experience results in reducing the bag lesions rate to zero [59].

Adverse events associated with bag use seem to be very rare. Numerous studies report no complications [63,64,65,82,87]. Only one study revealed minor complications in two of 33 patients (6%). In both cases, it was minimal bleeding from the trocar canal, which occurred during the extraction of the bag. It did not require any treatment other than standard coagulation with bipolar forceps [84]. A recently published systematic review on endobag use in gynecological laparoscopy did not find an increased complication rate when contained morcellation was performed [66]. The key aspects of contained morcellation are summarized in Table 1.

There is a paucity of research comparing different bag systems for contained morcellation. The only recently published study involved 33 women with uterine fibromas or adenomyosis, who were alternately allocated to a different retrieval bag system group [84]. The first container was manufactured of polyurethane and the second of polyurethan-coated nylon fibers. Both tested systems were dual-opening bags, that differed from each other in terms of dimensions, volume, mechanical properties of the materials, and the technique of introduction. According to the study results, the first system outperformed the second in terms of insertion of the bag, its positioning within the abdominal cavity, and removal. The possible explanation of this observation was that the first bag is delivered folded into a sleeve, which made insertion and positioning easier. On the other hand, significant differences regarding entering the optic trocar and establishing the pseudoperitoneum in favor of the second bag were observed. A probable cause was that the optic sleeve in the second system is shorter, which allows for insertion of the optic trocar without much trouble. Despite the above-listed minor differences, both tested retrieval bag systems proved to meet the criteria for safe and efficient contained morcellation.

The presented review has several limitations. There are no randomized control trials comparing laparoscopic uncontained and in-bag morcellation. Available studies are mainly retrospective and include limited numbers of patients. Finally, the definition of bag-related complications varies between studies, a fact which hinders proper interpretation.

## 4. Conclusions

Minimally invasive approaches, especially the laparoscopic technique, have significantly changed contemporary gynecology and are currently considered the “gold standard” in uterine fibroids surgery. Power morcellators were developed as a response to the requirement for special techniques to remove the specimen from the abdominal cavity inherent in the use of minimally invasive access. A 2014 FDA safety communication discouraged the routine use of open morcellation, given concerns for iatrogenic dissemination of a previously undiagnosed malignancy. Despite the unresolved issue of the occurrence and significance of this phenomenon, and even existing doubts whether a myoma can develop or transform into a sarcoma, all possible measures must be taken to optimize patient safety. Contained morcellation is considered to be one such solution that reduces the potential intraabdominal spread of undetected malignancy and myometrial cells, while simultaneously maintaining the benefits of a minimally invasive surgical approach. During the last decade, an intensive development of contained morcellation systems was observed—from single-port to multiport retrieval bags. Research has revealed positive learning curve effects, resulting in a reduction in the time associated with bag use and an increase in the size of the specimen successfully subjected to in-bag morcellation. The use of containment systems did not increase the rate of perioperative complications. However, it is essential to remember that in-bag morcellation is just one of the aspects of protecting patient safety. Qualification for laparoscopic uterine fibroids surgery with contained morcellation should be preceded by meticulous preoperative diagnosis, careful consideration of the advantages and risks of the planned approach, and thorough patient counseling. Further clinical trials are needed to more precisely determine the impact of the use of containment bags on the course of the operation as well as its effectiveness in the prevention of intraperitoneal tissue dissemination.

## Figures and Tables

**Table 1:** A summary of the key aspects of contained laparoscopic morcellation.

Types of containment bag	One-port
	Two-port
	Multiport
Volume	1.6–4.0 L
Weight of specimen subjected to contained morcellation	18–2805 g
Additional time related to bag use	7.5–26.5 min
Total surgical time	Comparable or shorter than without a bag
Intraoperative bag damage	6%
Adverse events related to bag use	0–6%
Overall complications rate	Comparable to procedures with open morcellation

## Data Availability

Data sharing not applicable—no new data generated.
